# From Detection to Prediction: Advances in m6A Methylation Analysis Through Machine Learning and Deep Learning with Implications in Cancer

**DOI:** 10.3390/ijms26146701

**Published:** 2025-07-12

**Authors:** Ruoting Jin, Quan Zou, Ximei Luo

**Affiliations:** 1 Institute of Fundamental and Frontier Sciences, University of Electronic Science and Technology of China, Chengdu 611731, China; 202422210332@std.uestc.edu.cn (R.J.); zouquan@nclab.net (Q.Z.); 2 Faculty of Applied Sciences, Macao Polytechnic University, Macau 999078, China

**Keywords:** N6-methyladenosine, machine learning, deep learning, cancer

## Abstract

N6-methyladenosine (m6A) represents the most common and thoroughly investigated RNA modification and exerts essential functions in regulating gene expression through influencing the RNA stability, the translation efficiency, alternative splicing, and nuclear export processes. The rapid development of high-throughput sequencing approaches, including miCLIP and MeRIP-seq, has profoundly transformed epitranscriptomics research. These techniques facilitate the detailed transcriptome-wide profiling of m6A modifications, shedding light on their crucial roles in diverse biological pathways. This review comprehensively examines the identification, mechanisms of regulation, and functional consequences of m6A modifications. It emphasizes their critical roles in physiological contexts, encompassing immune function, neuronal development, and the differentiation of stem cells. Additionally, the review discusses the contributions of m6A dysregulation to pathological conditions, including cancer, neurodegenerative diseases, and disorders of metabolism. We also discuss the development and application of machine-learning algorithms for m6A site prediction, emphasizing the integration of sequence-based, structural, and evolutionary conservation features to enhance the predictive accuracy. Furthermore, the potential of applying the findings from m6A research in precision medicine and drug development is examined. By synthesizing the current knowledge and emerging trends, this review aims to provide a comprehensive understanding of m6A biology and its translational potential, offering new perspectives for future research and therapeutic innovation.

## 1. Introduction

RNA methylation, particularly N6-methyladenosine (m6A), represents a pivotal RNA modification within the field of epitranscriptomics, recognized for its abundance and regulatory significance. m6A modifications substantially impact gene expression by influencing RNA splicing, nuclear export, the translation efficiency, and the RNA stability [1]. With recent technological breakthroughs, such as MeRIP-seq [2] and miCLIP [3], research on m6A has advanced significantly, enabling the comprehensive, high-resolution profiling of these modifications across entire transcriptomes and thus deepening the understanding of their biological roles.

This technological evolution has profoundly driven advancements in epitranscriptomics studies. The initial identification of m6A modifications utilized techniques such as high-performance liquid chromatography (HPLC) [1]. However, contemporary sequencing technologies now allow for transcriptome-wide mapping with improved throughputs and precision, thereby enhancing the granularity and scope of m6A research. Notably, the emergence of third-generation sequencing platforms, such as Pacific Biosciences’ SMRT sequencing platform and those produced by Oxford Nanopore Technologies, has further revolutionized the field by enabling the direct detection of m6A on native RNA molecules, providing unprecedented insights into isoform-specific modifications and their roles in long non-coding RNAs.

The regulation of m6A modifications involves intricate networks that include RNA-binding proteins such as the YTHDF protein family, alongside demethylases, particularly FTO and ALKBH5 [4,5]. These complex interactions are instrumental in modulating crucial physiological processes, including immune responses, neural development, stem cell differentiation, and cellular stress responses [6,7]. Furthermore, the dysregulation of m6A modifications has been closely linked with a variety of pathological states, notably cancer, neurodegenerative disorders, and metabolic syndromes [8,9]. Illustratively, the aberrant activities of m6A-regulatory enzymes like METTL3 and FTO have emerged as potential therapeutic targets in acute myeloid leukemia [10,11].

The growing depth of understanding regarding m6A modification mechanisms has facilitated the development of sophisticated machine learning algorithms designed for the precise prediction of m6A sites. These computational tools integrate diverse features, encompassing RNA sequence patterns, structural characteristics, and evolutionary conservation, thereby markedly enhancing the prediction reliability [12,13,14,15,16]. However, the inherent black-box nature of many complex deep learning models often poses challenges for biological interpretation, underscoring the critical need for methods that enhance models’ interpretability and facilitate the identification of key biological features [17]. The future integration of these predictive models with multi-omics datasets and rigorous experimental validation is anticipated to significantly advance precision medicine, therapeutic development, and complex disease analyses [18,19,20,21]. In this review, we systematically evaluate the recent discoveries related to m6A modifications, elucidating their regulatory mechanisms and functional roles in both gene expression regulation and disease pathogenesis. A detailed comparative analysis of various sequence-based and machine learning prediction methodologies is presented, alongside an exploration of their respective performance metrics, including a critical discussion on the role of variable selection methods in enhancing biological interpretability. Finally, we highlight the potential clinical applications of m6A research, particularly in precision medicine and pharmacological innovation, offering novel insights and outlining promising directions for subsequent studies.

## 2. Discovery of m6A Methylation

The initial identification of m6A modifications in mammalian mRNA dates back to 1974 [1,22], when Desrosiers and colleagues employed high-performance liquid chromatography (HPLC) to detect the presence of these modifications. This seminal finding established m6A as a prevalent RNA modification predominantly occurring on messenger RNAs (mRNAs) and long non-coding RNAs (lncRNAs) [23]. Subsequent research has elucidated the significant roles of m6A in modulating diverse aspects of RNA biology, including its stability, translation efficiency, splicing dynamics, and nuclear transport.

**Figure 1:**
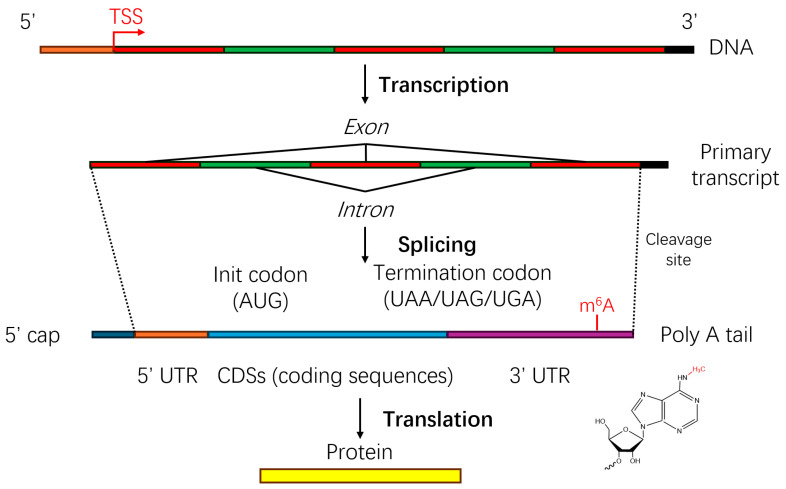
Schematic overview of the mRNA m6A methylation distribution within the eukaryotic gene expression pathway. Figure 1 provides a schematic overview of the transcription and translation processes, illustrating the typical sites of N6-methyladenosine (m6A) modification within mRNA. Following transcription from DNA, the primary transcript undergoes splicing to remove introns and join exons, resulting in mature mRNA with defined untranslated regions (UTRs) and coding sequences (CDSs). The m6A modification frequently occurs in the 3’ untranslated regions (3’ UTRs) and near stop codons, influencing the RNA stability, the translation efficiency, and alternative splicing.

Distinct localization patterns characterize m6A modifications within RNA molecules. These modifications frequently concentrate within specific transcript regions, notably the 5’ untranslated regions (5’UTRs) and 3’ untranslated regions (3’UTRs), particularly adjacent to stop codons and polyadenylation (polyA) signals, as shown in Figure 1. Such strategic positioning underscores their regulatory influence over the RNA stability, translational regulation, and nuclear–cytoplasmic export processes. Furthermore, m6A modifications commonly localize near exon–intron boundaries, actively participating in alternative splicing by modulating RNA secondary structures or recruiting specific splicing factors, thus contributing to transcript diversity. Sequence specificity studies have identified a conserved RNA motif associated with m6A methylation, RRACH [2,24,25,26,27], where R denotes a purine nucleotide, A represents the methylated adenosine, C is cytosine, and H is any nucleotide except guanine. This motif exhibits remarkable conservation across multiple species, highlighting its critical evolutionary significance.

In recent years, with the deepening of m6A modification research, increasing evidence has highlighted its important role in disease development. Abnormalities in m6A modification are closely associated with cancer [8,28], neurodegenerative diseases, and metabolic disorders. The discovery of the m6A demethylase encoded by the FTO gene further underscores the importance of m6A modification in physiological processes. These findings provide new perspectives for the development of future therapeutic strategies and lay a solid foundation for epitranscriptomics research.

## 3. Regulatory Mechanisms of m6A

The regulatory mechanisms governing m6A modification involve complex interactions among various RNA-binding proteins and demethylases, highlighting its crucial role in post-transcriptional control [4,29]. The dynamic and reversible nature of m6A enables it to finely regulate RNA metabolism at multiple regulatory stages. Among the key protein players are members of the YTHDF protein family, which exhibit diverse functional roles extending beyond merely signaling RNA degradation [30]. YTHDF2 primarily mediates mRNA degradation by recognizing and binding m6A-modified RNAs, subsequently recruiting the CCR4-NOT complex to initiate RNA deadenylation and decay. In contrast, recent evidence suggests that YTHDF1 and YTHDF3 demonstrate distinct properties involving phase separation, leading to the formation of membraneless compartments. This spatial organization mechanism plays a critical role in regulating translation under conditions of cellular stress [31].

Besides affecting RNA degradation pathways, m6A modifications enhance the RNA stability through interactions with other specific RNA-binding proteins, notably the IGF2BP protein family, protecting RNAs from degradation [6]. Furthermore, m6A profoundly influences the translation efficiency via multiple mechanisms. Within the 5’ untranslated region (UTR), m6A modifications interact with the translation initiation factor eIF3, facilitating translation initiation. Additionally, the binding of YTHDF1 to m6A-modified RNAs promotes translation elongation through direct interactions with ribosomes and other translation factors.

m6A also significantly affects alternative splicing processes by altering RNA secondary structures or recruiting specific splicing regulators. For example, m6A modifications facilitate the recruitment of the splicing factor SRSF2, thus promoting the selective inclusion or exclusion of exons, generating diverse splice variants. Intronic m6A modifications similarly influence the splicing outcomes by modulating the binding affinities of various splicing factors through alterations in the RNA structural dynamics [32].

The role of m6A extends to the regulation of nuclear export processes. Specifically, m6A modifications recruit the nuclear export factor ALYREF, enhancing the export of RNA from the nucleus into the cytoplasm. This mechanism is crucial in modulating gene expression profiles at the cellular level. Additionally, demethylases such as FTO and ALKBH5 reverse m6A modifications, affecting RNA’s stability and translation. For instance, FTO-mediated demethylation typically prolongs the RNA half-life and enhances the RNA stability [5].

Collectively, these intricate regulatory mechanisms underscore the diverse and context-dependent functions of m6A modifications in RNA biology, with significant implications for physiological processes such as neurodevelopment, immune responses, and differentiation pathways. The continued exploration of these regulatory pathways promises to offer deeper insights into their involvement in various pathological states, including cancer and neurodegenerative diseases, presenting new therapeutic strategies for clinical intervention.

## 4. Detection Techniques for m6A

### 4.1. MeRIP-Seq and m6A-Seq

The MeRIP-seq (Methylated RNA Immunoprecipitation Sequencing) [2] and m6A-Seq methodologies integrate immunoprecipitation with high-throughput sequencing to profile m6A modifications throughout the transcriptome comprehensively. These approaches utilize antibodies specifically recognizing m6A to selectively enrich modified RNA fragments. The subsequent high-throughput sequencing of these enriched segments produces overlapping sequence data, manifesting as peaks, with their apexes indicative of probable m6A sites. A significant advantage of these methods lies in their mature protocols, robust throughput capabilities, and well-established analytical frameworks, enabling extensive profiling across diverse biological contexts. Nonetheless, their limited resolution, typically spanning approximately 100–200 nucleotides, restricts their capability for precise single-nucleotide localization. Furthermore, substantial quantities of RNA samples are required, which may limit their utility in scenarios with scarce biological material.

### 4.2. m6A-CLIP and miCLIP

m6A-CLIP (UV cross-linking and immunoprecipitation) and miCLIP (Methylation Individual-Nucleotide-Resolution Cross-Linking and Immunoprecipitation) [3] combine UV cross-linking and immunoprecipitation. UV light is used to cross-link RNA with m6A-binding proteins, followed by the enrichment of RNA fragments containing m6A modifications and high-throughput sequencing. These techniques utilize UV light to cross-link RNA with m6A-binding proteins (YTHDF family proteins), stabilizing the RNA–protein complexes.

By enriching cross-linked RNA fragments with m6A-specific antibodies and performing high-throughput sequencing, m6A-CLIP and miCLIP precisely locate m6A modification sites. These techniques are primarily used for the single-nucleotide-resolution mapping of m6A modifications, revealing the specific locations of m6A in RNA molecules and studying the interactions between m6A modifications and their binding proteins. The advantages of these techniques include their high resolution, providing a single-nucleotide resolution, and improved specificity and sensitivity achieved through cross-linking and immunoprecipitation. However, these techniques involve complex experimental procedures, long operation cycles, and high requirements regarding the sample quality and experimental conditions.

### 4.3. SCARLET

SCARLET (Site-Specific Cleavage and Radioisotope Labeling followed by Ligation-Assisted Extraction and Thin-Layer Chromatography) [33] is an m6A detection technique based on radioisotope labeling and thin-layer chromatography, capable of precisely locating single m6A modification sites. This technique uses specific enzymes to cleave and radioisotope-label m6A-modified RNA fragments, followed by thin-layer chromatography to separate and detect the labeled RNA fragments, precisely locating m6A modification sites. SCARLET is primarily used in research requiring a high resolution and specificity, providing a single-nucleotide resolution for precise m6A modification site mapping. Its advantages include its high resolution and specificity, with an improved detection specificity and sensitivity achieved through specific cleavage and labeling. However, SCARLET involves complex experimental procedures, long operation cycles, and the use of radioisotopes, requiring special attention to safety and handling.

### 4.4. scDART-Seq

scDART-seq (single-cell DART-seq) is a high-throughput m6A detection technology that operates at a single-cell resolution, enabling the study of the m6A modification heterogeneity across individual cells [34]. This technique builds upon DART-seq (Deamination Adjacent to RNA Modification Target Sequencing) [35]. The core principle of scDART-seq relies on the use of the APOBEC1-YTH fusion protein, which specifically recognizes m6A modification sites. The cytidine deaminase (APOBEC1) within the fusion protein converts adjacent cytosine (C) residues to uracil (U), generating C-to-U mutation signals during sequencing. These mutation signals enable the precise localization of m6A modification sites. The key advantage of scDART-seq is its single-cell resolution, which reveals the heterogeneity of m6A modifications across different cells. This capability offers new perspectives for understanding the role of m6A modifications in cell fate determination, development, and disease [36,37].

The scDART-seq workflow consists of several steps: single-cell isolation, fusion protein treatment, RNA extraction and reverse transcription, and high-throughput sequencing. Single-cell isolation techniques, such as microfluidics or fluorescence-activated cell sorting (FACS), are used to isolate individual cells in separate reaction systems. The APOBEC1-YTH fusion protein is then introduced into the cells, where it recognizes m6A modification sites and induces C-to-U mutations. RNA is extracted from the single cells and reverse-transcribed into cDNA [38,39]. High-throughput sequencing is performed on the cDNA to detect C-to-U mutation signals. Finally, bioinformatics analysis is used to identify m6A modification sites and assess the heterogeneity of the m6A modifications at the single-cell level.

The strengths of scDART-seq include its high resolution and ability to detect m6A modifications at the single-cell level, revealing dynamic changes and variability in the m6A modifications across individual cells. However, the technique involves complex experimental procedures, requires a long operation cycle, and demands a high sample quality and stringent experimental conditions.

### 4.5. Third-Generation Sequencing Technologies

The advent of third-generation sequencing technologies, particularly Pacific Biosciences’ Single-Molecule, Real-Time (SMRT) sequencing technology and Oxford Nanopore Technologies (ONT) products, has revolutionized genomics and transcriptomics by enabling the sequencing of individual long nucleic acid molecules [40]. Crucially, these platforms offer a paradigm shift for m6A detection by facilitating the direct analysis of native RNA molecules, thus circumventing the need for chemical treatment or fragmentation inherent in traditional short-read methods like m6A-seq or MeRIP-seq.

PacBio SMRT sequencing identifies m6A modifications by analyzing subtle kinetic changes in the DNA polymerase activity as it synthesizes a complementary strand across a modified RNA template [41]. Specifically, an m6A modification at an adenosine residue can alter the polymerase’s kinetics, leading to characteristic inter-pulse duration (IPD) and pulse width (PW) signals that are distinguishable from those of unmodified bases. This method provides high accuracy and long reads, allowing for isoform-specific m6A profiling and the detection of m6A in full-length transcripts. In parallel, Oxford Nanopore sequencing directly senses m6A by detecting distinct electrical current perturbations as an RNA molecule passes through a nanopore [42]. Each nucleotide, and indeed each specific modification, like m6A, generates a unique electrical signature. This direct approach offers the advantages of ultra-long reads, real-time data acquisition, and the potential to simultaneously detect multiple RNA modifications in a single transcript, providing an unprecedented view of the epitranscriptomic landscapes in long non-coding RNAs and full-length mRNAs. While these technologies offer unparalleled advantages in terms of their directness and resolution, challenges remain regarding their higher raw error rates (for ONT products, though these are continuously improving) and the development of robust, standardized bioinformatics pipelines for accurate m6A calling and quantification.

### 4.6. Comparison of m6A Detection Techniques

As summarized in Table 1, the current techniques for detecting m6A modifications vary in terms of their resolution, throughput, and complexity. MeRIP-seq provides transcriptome-wide coverage at a lower cost but has a limited resolution, typically between 100 and 200 nucleotides, making precise single-nucleotide localization challenging. Techniques such as m6A-CLIP and miCLIP offer high, single-nucleotide resolution suitable for precise mapping and RNA–protein interaction analysis; however, their experimental complexity, longer operational cycles, and stringent sample requirements can limit their applicability. SCARLET achieves high specificity and a nucleotide-level resolution, yet involves radioisotopic labeling, resulting in additional complexity and safety considerations. scDART-seq uniquely offers a single-cell resolution with a high throughput, effectively revealing intercellular heterogeneity but requiring rigorous experimental conditions and high sample quality. Selecting appropriate methods thus depends critically on the experimental goals, resource availability, and precision needed in m6A site detection.

## 5. Prediction Methods for m6A

### 5.1. Sequence-Based Methods

Sequence feature-based m6A prediction methods were among the earliest strategies and are some of the most widely applied [12]. These methods leverage the conserved sequence features of known m6A modification sites to build predictive models through statistical analysis, identifying potential m6A sites. m6A modifications typically occur in specific sequence contexts, most commonly the RRACH motif. Based on this feature, researchers can identify potential m6A modification sites [43]. By statistically analyzing the sequence features of known m6A modification sites, simple predictive models are constructed to scan the entire transcriptome sequence and identify sites that match the RRACH motif as potential m6A modification sites. Additionally, the PEA method uses position-specific propensity matrices to statistically analyze the nucleotide distribution around known m6A modification sites, building predictive models [44]. By constructing position-specific propensity matrices, the probability of m6A modification at each candidate site is calculated. For each candidate site, the nucleotide distribution in its surrounding sequence is compared with the propensity matrix of known m6A modification sites to predict whether it is an m6A modification site. Evolutionary conservation analysis is also a commonly used method, as m6A modification sites are often conserved during evolution [45]. By comparing homologous sequences across different species, conserved m6A modification sites can be identified. Multiple sequence alignment is used to recognize conserved m6A modification sites across species, and phylogenetic tree analysis is employed to calculate the evolutionary conservation score of candidate sites. This information, combined with the sequence features, enhances the accuracy of m6A modification site prediction.

### 5.2. Traditional Machine Learning-Based Methods

Classical machine learning methods have significantly contributed to enhancing the predictive accuracy for m6A modification sites, particularly suitable for medium-sized datasets characterized by high dimensionality. These techniques typically involve explicit feature extraction and selection procedures, predictive model construction, and rigorous performance evaluation [46,47].

As a convex optimization model, Support Vector Machines (SVMs) [48], commonly used in classification tasks, discriminate between modified and non-modified RNA sequences by determining the optimal separating hyperplanes in multi-dimensional feature spaces. Notable predictive models, such as iRNA-m6A, effectively integrate sequence motifs with other bioinformatics-derived attributes to achieve commendable accuracy [49]. A method named RAM-ESVM was developed for m6A site identification, integrating ensemble Support Vector Machine classifiers with some novel sequence features. Through rigorous benchmarking against *S. cerevisiae* transcriptomic datasets, this ensemble learner achieved commendable predictive accuracy [50]. Notably, when equipped with regularization terms such as the Ridge penalty (L2 norm) on their hinge loss function, SVMs also implicitly perform feature shrinkage, contributing to variable selection by reducing the influence of less important features and enhancing the model interpretability.

Random Forest (RF) is an ensemble learning method that constructs multiple decision trees and combines their predictions through majority voting to produce a final result, thereby achieving classification. In m6A modification prediction, Random Forest incorporates various features, such as sequence features, structural features, and evolutionary conservation, to make predictions. The SRAMP tool leverages the Random Forest algorithm to predict m6A modifications and has demonstrated high predictive accuracy [12]. A key advantage of RF is its intrinsic ability to perform variable ranking and feature importance assessment, allowing for the identification of the most discriminative features contributing to the prediction.

Similarly to Random Forest, XGBoost also provides robust feature importance scores, which can be directly utilized for variable selection and to understand the relative contribution of different features to the model’s predictions. The HSM6AP model achieves the high-precision prediction of N6-methyladenosine sites in human RNA by innovatively integrating sample weighting strategies, multi-dimensional feature fusion, and an XGBoost-based ensemble learning framework [51]. Its superior performance, rigorously validated using cross-validation and multiple independent datasets, surpasses existing state-of-the-art tools, offering a robust computational platform to elucidate the biological functions of m6A modifications and their regulatory roles in disease mechanisms.

### 5.3. Deep Learning-Based Methods

**Figure 2:**
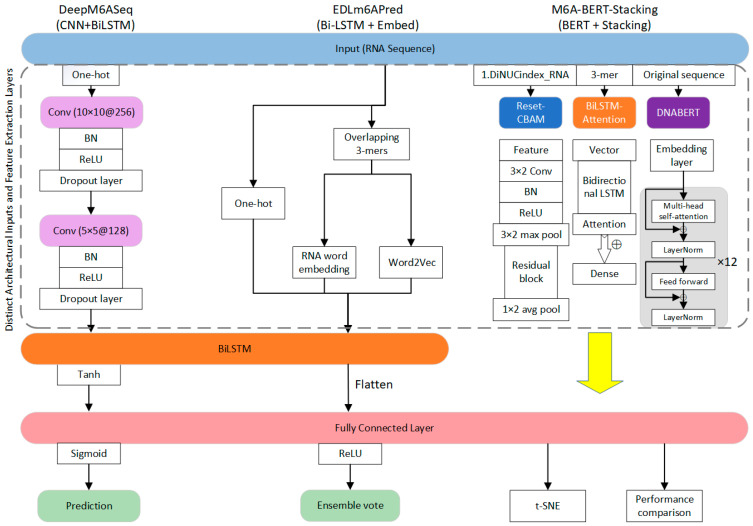
Architectural comparison of the deep learning models for m6A site prediction, highlighting their diverse feature extraction and integration strategies. Figure 2 illustrates the architectures and ensemble process of three distinct m6A site prediction models: DeepM6ASeq, EDLm6APred, and M6A-BERT-Stacking. DeepM6ASeq combines Convolutional Neural Networks (CNNs) with bidirectional Long Short-Term Memory (BiLSTM) networks, EDLm6APred integrates RNA word embeddings with Word2Vec using a BiLSTM model, and the M6A-BERT-Stacking model leverages a hybrid feature extraction approach consisting of ResNet-CBAM, BiLSTM-Attention, and DNABERT. Features extracted by these models are eventually integrated via a fully connected layer to generate the final prediction outcomes. For a comparative overview of the key features across various other m6A prediction tools, please refer to Table 2.

Deep learning algorithms have demonstrated excellent performance in m6A modification prediction, particularly excelling in handling large-scale datasets and complex nonlinear problems. These algorithms significantly improve the prediction accuracy for m6A modification sites by constructing multi-layer neural networks to automatically extract features. As depicted in Figure 2, we integrated three prediction models, DeepM6ASeq, EDLm6APred, and M6A-BERT-Stacking, to enhance the prediction accuracy for m6A modification sites in RNA sequences. These models employed different deep learning architectures to capture diverse hierarchical and contextual information. After the extraction and fusion of the features from each approach, the integrated representation passed through a fully connected layer to yield the final prediction. This ensemble strategy capitalized on the complementary strengths of varied deep learning paradigms, effectively improving the robustness and accuracy of the overall prediction framework [52,53,54]. However, its high computational costs and extensive data and resource requirements for training remain challenges.

Convolutional Neural Networks (CNNs) have demonstrated notable effectiveness in capturing localized sequence features, as exemplified by the DeepM6ASeq framework [13], which is recognized for its precision in identifying m6A sites through convolutional processing. Through the integration of convolutional, pooling, and fully connected layers, CNNs are capable of autonomously extracting meaningful local patterns from input data. In the domain of m6A site prediction, these networks form layered architectures that not only capture sequence-specific signals but also incorporate broader bioinformatics attributes. DeepM6ASeq, in particular, leverages this architecture to attain a commendable prediction accuracy in identifying m6A modifications [13]. Additionally, Gene2vec offers a highly accurate approach for detecting m6A sites in mammalian transcripts. This model combines subsequence embeddings with deep learning by segmenting RNA sequences into trinucleotide “semantic units” and applying a Word2vec-inspired natural language processing technique. This enables the model to discern contextual patterns and semantic equivalence within RNA sequences [55]. In another contribution, the iRicem6A-CNN model utilized a dinucleotide one-hot encoding strategy alongside a CNN to predict the N6-methyladenine modifications in rice DNA. By accounting for the nucleotide context surrounding the target sites, it enhanced the predictive performance and highlighted the value of higher-order sequence representation in epigenomic research [56]. These methods have demonstrated the superiority of CNNs in sequence extraction and their effectiveness in m6A prediction.

Long Short-Term Memory (LSTM) [57], an improved Recurrent Neural Network (RNN) [58], introduces gating mechanisms to address the vanishing and exploding gradient problems associated with traditional RNNs when handling long sequences. In m6A modification prediction, LSTM constructs networks that capture the long-term dependencies in RNA sequences. The EDLm6APred tool employs bidirectional LSTMs combined with word embedding algorithms to predict m6A modification sites [59]. DeepM6ASeq-EL is an ensemble learning model that integrates LSTM and CNN architectures to predict m6A sites in human mRNA [60]. By combining five LSTM-CNN subnetworks using a hard voting strategy, the model leverages diverse sequence context features, while a comparative analysis of the encoding methods highlights the superior generalizability of single-nucleotide encoding. Transformer-based models, which leverage self-attention mechanisms to capture contextual information within sequences, have achieved great success in natural language processing. In m6A modification prediction, the M6A-BERT-Stacking tool integrates BERT and stacking strategies to predict tissue-specific m6A modification sites [61]. Collectively, these models demonstrate the growing effectiveness of sequential deep learning architectures, particularly LSTM and Transformer-based approaches, in capturing both local and global contextual features essential for accurate m6A modification prediction.

### 5.4. Other Prediction Methods

In addition to sequence-based and machine learning methods, other prediction methods have also been applied in m6A modification research. For example, methods that utilize molecular spatial structures and physicochemical properties can capture the complex relationships between RNA molecules by constructing molecular graphs and residual networks, thereby improving the prediction accuracy for m6A modification sites [62]. Furthermore, multi-omics data integration methods combine genomic, transcriptomic, and proteomic data to build more comprehensive predictive models, revealing the regulatory networks and functional mechanisms of m6A modification [63]. One study introduced DeepM6APred [64], a novel computational tool that integrates deep feature representations learned by a deep belief network with traditional handcrafted features to significantly enhance the prediction accuracy of N6-methyladenosine sites in RNA, achieving state-of-the-art performance and providing an accessible web server for practical applications.

Beyond these categorized approaches, a wide array of computational tools has been developed to implement and facilitate m6A prediction across various contexts. To provide a comprehensive overview of the diverse landscape of available software for m6A research, including the specific prediction tools discussed throughout the preceding sections, Table 2 provides an overview of various computational tools and software developed for m6A modification research, summarizing their main functionalities, underlying programming languages or platforms, key architectural features, and availability information. This consolidated resource aims to guide researchers in selecting appropriate computational tools based on their specific research needs and experimental setups.

### 5.5. Interpretability and Variable Selection in m6A Prediction

While sophisticated machine learning and deep learning models have achieved remarkable predictive performance in m6A research, their “black-box” nature, particularly in complex deep neural networks, often limits the direct biological interpretation of their predictions. Understanding why a model makes a specific prediction and identifying the underlying influential biological features are paramount for advancing both fundamental biological discoveries and clinical translation. To address this, variable selection emerges as a critical computational strategy, aiming to identify a subset of highly relevant features from high-dimensional omics data that are the most strongly associated with m6A modifications or cancer phenotypes, thereby significantly enhancing the model interpretability and ability to gain biological insights.

Variable selection methods can broadly be categorized based on their relationship with the supervised learning process. In the realm of supervised learning, classical penalization methods are particularly effective. For instance, in models like Support Vector Machines (SVMs), which minimize a hinge loss function, the inclusion of regularization terms such as the Ridge penalty (L2 norm) intrinsically promotes feature shrinkage, thereby contributing to the implicit selection of relevant variables. As illustrated in Figure 2 in a study by Wu et al. [17], such regularization schemes are consistent with widely adopted regularized variable selection frameworks. Similarly, tree-based ensemble models like Random Forest and XGBoost possess a natural capability for variable ranking. These methods inherently quantify the importance of each feature by assessing its contribution to node impurity reduction (e.g., the Gini impurity or information gain) or split decisions across the ensemble of trees. This allows for the identification and prioritization of the most discriminative features in the dataset, effectively serving as a powerful variable selection mechanism.

Beyond their direct application, variable selection methods play a crucial role complementary to deep learning approaches. They can be effectively employed as a preprocessing step, filtering and selecting an informative subset of features from high-dimensional omics data before feeding them into deep learning models. This not only reduces the input dimensionality, potentially mitigating overfitting in smaller datasets, but also ensures that the deep learning model focuses on the most biologically salient information. Alternatively, variable selection techniques can serve as post hoc interpretation tools, aiding in the explanation of complex patterns learned by deep neural networks by identifying which input features are the most heavily weighted or activated by the model, thereby offering insights into the deep model’s “decision-making” process and moving towards biological understanding.

### 5.6. Performance Comparison and Evaluation of Existing Tools

In m6A modification prediction, deep learning algorithms and traditional machine learning algorithms each have their advantages and disadvantages. Deep learning algorithms, such as CNNs, Bi-LSTM, and BERT combined with stacking strategies, demonstrate exceptional performance by constructing multi-layer neural networks to automatically extract features. For instance, DeepM6ASeq leverages a CNN to predict m6A modifications, enabling the automatic extraction of local features from RNA sequences. It exhibits strong feature extraction capabilities and high predictive accuracy, making it suitable for large-scale data. However, CNNs are computationally expensive and require substantial data and computational resources for training. EDLm6APred employs Bi-LSTM combined with word embedding algorithms to capture the contextual information and long-term dependencies in RNA sequences, thereby improving the accuracy of m6A site prediction. While Bi-LSTM performs well in handling long sequences, it also faces high computational costs. CLSM6A uses multi-layer neural networks to automatically extract single-nucleotide-resolution features, making it suitable for making high-resolution m6A modification predictions with high predictive accuracy. M6A-BERT-Stacking combines BERT and stacking strategies to capture the contextual information in RNA sequences, enhancing the overall prediction performance through stacking and excelling in tissue-specific m6A modification predictions. DLm6Am, utilizing multi-layer neural networks, automatically extracts sequence and chemical features, demonstrating a high generalization ability and predictive accuracy for the efficient recognition of m6Am modifications.

In contrast, traditional machine learning algorithms, such as SVMs, RF, k-nearest neighbors (KNN), Naive Bayes (NB), and Logistic Regression (LR), perform well on small- to medium-sized datasets and high-dimensional data. These algorithms exhibit high precision and generalization abilities. The performance metrics of common m6A prediction tools are shown in Table 3. As these tools were evaluated using diverse datasets and validation strategies, the performance metrics presented in this table should be interpreted as general trends, acknowledging that the specific performance can vary significantly with different evaluation conditions. Acknowledging this, and given the absence of variances or standard deviations in the original reported data, the performance metrics in this table are represented as single reported values. Therefore, to facilitate more comprehensive and reliable comparisons, incorporating measures of variance will be crucial in future benchmarking efforts, as their omission may otherwise lead to biased or incomplete assessments of the model performance. An SVM achieves classification by finding an optimal hyperplane in a high-dimensional space to maximize the margins between classes. It is well-suited for small- to medium-sized datasets and can handle nonlinear classification problems. However, SVMs have high computational complexity when used with large-scale datasets, and their performance is sensitive to kernel functions and the parameter selection. RF builds multiple decision trees and combines their predictions through voting, achieving high accuracy and strong feature selection capabilities. While it handles high-dimensional data effectively, RF has high computational complexity and limited interpretability. KNN formulates predictions by calculating the distance between new samples and training samples, finding the k-nearest neighbors, and determining the class based on these neighbors. It is suitable for use with small-scale datasets but suffers from high computational costs with large-scale data. NB, based on Bayes’ theorem, makes predictions by calculating the conditional probabilities between features and classes. It is computationally fast and performs well on small-scale datasets but assumes feature independence, which may not hold for all datasets. LR estimates the relationship between features and classes by maximizing the likelihood function. It has strong interpretability and is suitable for use with linearly separable datasets but performs poorly on nonlinear problems. This comparative performance evaluation of several widely used m6A prediction tools highlights substantial variations across the different methods. Thus, selecting an appropriate computational method requires the consideration of the specific performance requirements, available computational resources, and targeted application scenarios.

While advanced prediction models have demonstrated considerable promise in m6A research, it is crucial to acknowledge their inherent limitations and the challenges they face, particularly in the context of cancer omics. A prominent issue is overfitting in small datasets: despite the high dimensionality of cancer omics data, often comprising tens of thousands of genes or features, the available sample sizes (patient numbers) are frequently limited. Complex machine learning algorithms, especially deep learning models with their numerous parameters, are highly prone to overfitting when trained on such constrained datasets. This phenomenon leads to models that perform exceptionally well on the training data but exhibit significantly degraded predictive power and a poor generalization ability when applied to unseen or real-world clinical data, rendering their predictions unreliable. Deep learning models fundamentally require vast amounts of data to effectively learn intricate patterns and distinguish signals from noise; consequently, insufficient data often leads to them inadvertently learning the specific noise and random errors within the training set, rather than the underlying biological regularities.

Another critical concern pertains to the black-box nature and lack of interpretability inherent in many deep learning models. While these models excel at prediction tasks, discerning how they arrive at a particular prediction—identifying which input features contributed most significantly to the final outcome or understanding the model’s internal decision logic—remains exceptionally challenging. In the biomedical domain, this deficiency in interpretability presents a substantial obstacle, as clinicians and researchers require not only the “what” (the prediction result) but also, crucially, the “why” (the underlying biological mechanisms). To address these challenges, recent sophisticated deep learning frameworks, such as cfMethylPre, have leveraged strategies like deep transfer learning and integrated information from large language models to enhance feature representation, improve the predictive accuracy with limited sample sizes, and provide greater biological interpretability in cancer detection [65]. This opacity hinders the discovery of novel biological insights, complicates the identification of robust biomarkers, impedes clinical translation by eroding the trust in models’ predictions, and makes it difficult to detect instances where a model might be making predictions based on spurious or non-relevant features. Consequently, when facing limited amounts of data, alternative strategies focusing on robust feature engineering and traditional machine learning models often prove more effective, as exemplified by the SBSM-Pro model, which leverages Support Vector Machines and sophisticated amino acid grouping to successfully address small-sample problems in protein sequence classification [66].

Furthermore, these models often suffer from limitations in their generalizability due to data heterogeneity. Prediction models frequently demonstrate diminished performance when deployed across different datasets or research institutions. This variability stems from multifaceted data heterogeneity, including differences in the sequencing platforms, sample processing protocols, and batch effects and diverse genetic backgrounds or clinical characteristics among patient cohorts. Such inconsistencies can lead a model trained on one dataset to perform poorly when applied to another, thereby restricting its universal applicability and clinical utility. Beyond technical variations, the quality and representativeness of training data are paramount, as the model performance is highly dependent on these factors. Noise, erroneous labels, missing values, or systemic biases within the data can directly compromise the learning process and final model’s efficacy. For instance, if a training dataset is biased—comprising samples predominantly from a specific ethnic group or clinical subtype—the model may inadvertently learn and perpetuate these biases, leading to suboptimal or even discriminatory predictions in other populations or contexts. Consequently, ensuring the availability of large-scale, high-quality, and unbiased m6A and cancer omics datasets is fundamental to advancing the model performance. Lastly, the computational resource demands of complex deep learning models are considerable, typically necessitating high-performance graphics processing units (GPUs) and substantial memory for both training and deployment. This requirement can pose a significant barrier for laboratories with limited research infrastructure, thereby restricting the widespread adoption and exploration of advanced computational approaches. Future research will aim to optimize existing algorithms, integrate multi-omics data, and incorporate experimental validation to develop more precise and efficient m6A modification prediction tools, providing new insights into the functional study of m6A modifications.

## 6. Applications of m6A in Cancer and Other Diseases

Recently, m6A methylation, as a prevalent RNA modification, has increasingly attracted attention within cancer research due to deeper insights being obtained into its regulatory mechanisms and the advancement of analytical technologies [67,68]. Substantial progress has been achieved in understanding its roles in cancer and other pathologies. This section discusses m6A’s potential for use as a diagnostic biomarker, explores therapeutic interventions targeting m6A pathways, and examines its predictive value regarding cancer prognosis.

### 6.1. Potential of m6A for Use as a Biomarker

Distinct alterations in the m6A methylation patterns observed across various cancers have positioned m6A as a potential biomarker. Studies demonstrated significant differences in the m6A methylation levels between cancer types, and these differences correlated closely with the tumor progression, pathological grading, and clinical outcomes [69]. For instance, decreased m6A levels in hepatic cancer tissues are indicative of increased malignancy and poorer prognostic outcomes. Additionally, enzymes involved in m6A modifications, such as METTL3 and FTO, have exhibited abnormal expression in diverse cancers [70]. Such findings underscore the potential utility of m6A modifications and the associated enzymatic expression profiles as novel biomarkers for early cancer detection, disease progression monitoring, and prognostic evaluation.

### 6.2. m6A-Targeted Therapeutic Strategies

The dynamic and reversible nature of m6A modification provides new targets for cancer therapy. The development of drugs targeting m6A-modifying enzymes has become a hot research topic. For example, inhibiting METTL3 expression reduces the proliferation and survival of leukemia cells, while FTO inhibitors show therapeutic potential for acute myeloid leukemia [10,11]. Additionally, regulating m6A modification to influence the mRNA stability and translation efficiency can control the expression of specific oncogenes or tumor suppressor genes, thereby inhibiting tumor growth. Combination therapies, such as integrating m6A-targeted therapy with immunotherapy, also show promising applications [70]. These studies provide a theoretical and experimental basis for developing new cancer treatment strategies based on m6A modification. It was demonstrated in a study that N6-methyladenosine-associated long non-coding RNAs were significantly correlated with the clinical staging [71], survival prognosis, and tumor progression in esophageal cancer by modulating the tumor microenvironment, immune cell infiltration, CD4 memory resting T cells and M2 macrophages, and activity of critical signaling pathways, focal adhesion, and riboflavin metabolism. These m6A-lncRNAs served as independent prognostic biomarkers, validated through multivariate Cox regression and nomogram analyses. Furthermore, this research delineated the regulatory networks of m6A-lncRNAs in esophageal carcinogenesis and established a robust prognostic signature comprising five key lncRNAs. The study not only elucidated mechanistic insights into m6A-mediated epigenetic regulation but also provided novel biomarkers and a theoretical foundation for advancing clinical prognostic evaluation and targeted therapeutic strategies in esophageal cancer.

### 6.3. Correlation Between m6A and Cancer Patient Prognosis

The association between the m6A methylation levels and clinical prognosis in cancer patients is becoming increasingly evident. Numerous investigations have confirmed that variations in m6A methylation serve as independent prognostic indicators of patient survival across different cancers [72]. For instance, elevated m6A levels in breast cancer correlate positively with favorable outcomes, whereas diminished m6A methylation in colorectal cancer patients correlates negatively with the survival duration. Moreover, the expression of m6A-regulatory enzymes, including METTL14, significantly correlates with the patient prognosis. These insights contribute to the formulation of prognostic models predicated on m6A modifications, thereby enhancing personalized cancer management and precision medical interventions.

### 6.4. Applications in Brain-Related Topics and Neurological Disorders

While the application of m6A occurrence prediction methods has predominantly focused on cancer-related topics, the growing understanding of m6A’s pivotal roles in neurobiology necessitates an expansion of computational investigations into brain-related fields and neurological disorders. m6A methylation is highly abundant in the central nervous system (CNS), with its overall levels increasing from the embryonic to adult brain stages, suggesting its critical involvement in normal brain development and function [7]. Studies have elucidated that m6A modifications precisely regulate key aspects of mammalian cortical neurogenesis, impacting the proliferation and differentiation of neural stem/progenitor cells (NPCs). For instance, the depletion of m6A methyltransferases like Mettl14 in embryonic mouse brains extends the cell cycle of radial glia cells and prolongs cortical neurogenesis into the postnatal stages, indicating m6A’s role in the temporal control of neural cell fate [73]. Similarly, the m6A demethylase FTO is dynamically expressed in adult neural stem cells and neurons, and its loss can lead to a decreased brain size, and impaired learning and memory in mice [74]. Furthermore, m6A signaling has been shown to regulate human cortical neurogenesis in forebrain organoids, and human-specific m6A landscapes are enriched in transcripts related to brain disorder risk genes. These profound biological implications underscore the immense potential for applying m6A prediction methods to decipher the epitranscriptomic regulatory mechanisms underpinning brain development and neurological diseases.

Despite this clear biological significance, and while some promising computational advances have begun to address m6A prediction in brain tissues, such as the GR-m6A model, which effectively predicts m6A sites in mammalian brains using molecular graphs and residual network architectures [62], the comprehensive application and in-depth biological interpretation of these prediction models specifically for understanding the pathogenesis of neurological disorders or complex neural processes remain an underexplored area. The inherent complexity and heterogeneity of brain tissues, encompassing diverse cell types and intricate neuronal circuits, demand highly sophisticated models capable of capturing tissue- and cell-specific m6A patterns. The current prediction tools, while effective in certain contexts, may encounter limitations when dealing with the nuanced m6A landscapes of the brain, where the modifications might exhibit subtle kinetic or stoichiometric variations. Furthermore, the availability of large-scale, high-resolution m6A sequencing data from diverse brain regions, developmental stages, and neurological disease cohorts—especially for human samples—remains more constrained than that of cancer datasets. This data scarcity can impede the training and rigorous validation of advanced machine learning and deep learning models, potentially leading to issues such as overfitting or limited generalizability. Therefore, future efforts in this direction must prioritize not only the development of advanced algorithms tailored to the unique characteristics of neuronal m6A but also the generation of comprehensive and high-quality brain-specific epitranscriptomic datasets, including those for animal models, to fully harness the diagnostic and therapeutic potential of m6A in neurological health and disease.

## 7. Future Directions and Conclusions

### 7.1. Improving m6A Prediction Accuracy Using Multiple Methods

With the continuous advancement of bioinformatics, improving the precision of predicting m6A methylation sites remains a central objective in this field. A promising approach involves integrating diverse methodologies. A key strategy lies in the integration of multi-omics datasets, such as genomics, transcriptomics, proteomics, and metabolomics datasets. By synthesizing information from these different biological layers, researchers can gain a more nuanced understanding of the regulatory roles and functional dynamics underlying m6A modifications, thereby enhancing the reliability and depth of computational models. Furthermore, incorporating spatial transcriptomics and other spatial omics technologies will be crucial for understanding m6A’s context-dependent roles within heterogeneous cellular microenvironments, particularly in tumors, thereby providing a more effective means for biological mechanistic interpretation. Innovative algorithmic designs will also play a pivotal role. While traditional machine learning techniques offer certain strengths, they may fall short in effectively modeling complex biological systems. Consequently, emerging paradigms like deep learning and reinforcement learning are expected to provide more robust solutions. These advanced methods are better equipped to capture nonlinear associations and intricate sequence patterns, contributing to greater resilience and accuracy in predictive modeling. Furthermore, empirical verification using experimental data remains an indispensable part of model development. By systematically validating computational predictions against laboratory results, models can be iteratively refined and calibrated, thus improving both their biological relevance and practical applicability.

### 7.2. Development of and Demand for Customized Tools for Different Bioinformatics Applications

As m6A research deepens, the different fields in which there is a demand for bioinformatics tools from researchers are becoming increasingly diverse. Therefore, developing customized tools for different applications will be an important future direction. The development of personalized tools will meet specific research needs. For example, m6A prediction tools for specific cancer types will help researchers gain deeper insights into the molecular mechanisms of cancer, providing strong support for precision medicine. The establishment of multifunctional platforms will improve research efficiency. A multifunctional bioinformatics platform integrating prediction, analysis, and visualization will greatly simplify research processes, enabling researchers to obtain the required information more quickly and conveniently. User-friendliness is also an important consideration in tool development. By optimizing interface designs and providing detailed user guides and tutorials, we can make these advanced tools accessible to non-expert users, thereby broadening their application scope.

### 7.3. Data Sharing and Standardization

Establishing a robust data-sharing mechanism will accelerate scientific discovery by encouraging researchers to share m6A-related data, promoting collaborative research. Data sharing also helps avoid redundant research and improves research efficiency. Crucially, promoting reproducible research in m6A prediction necessitates the development of unified standardization processes for tool development and evaluation. This includes standardizing data collection, processing, and analysis procedures, as well as establishing user-friendly, high-reproducibility bioinformatics analysis platforms and cross-laboratory standardized workflows to facilitate broader adoption and validation. Strengthening data security management is crucial during data sharing to protect patients’ privacy and intellectual property rights. By establishing strict data security management systems, we can ensure the safety and legality of data sharing.

### 7.4. Conclusions

In summary, this review systematically synthesizes the significant progress made in m6A research, with a particular emphasis on the evolution and application of computational prediction methods in the context of cancer studies. By thoroughly evaluating various sequence-based, machine learning, and deep learning algorithms, we have elucidated their respective strengths and current limitations for m6A site prediction. A key contribution of this review lies in identifying the current bottlenecks and unique challenges associated with the application of these predictive models, especially regarding their interpretability and generalizability within the complex landscape of cancer omics data. Machine learning-based m6A modification site prediction technologies urgently need further development and validation. The stable prediction capabilities of these algorithms across different datasets and biological systems have not been fully validated. Combining high-throughput sequencing and immunoprecipitation data to validate machine learning prediction results is an efficient method. Additionally, observing genome-wide changes by altering the RNA methylation states, such as by knocking out related enzymes or through pharmacological inhibition, provides new perspectives for studying the functions of m6A modification. m6A modification often interacts with multiple RNA sites, and knocking out a single related enzyme may not fully reveal its mechanisms. Therefore, identifying key proteins interacting with m6A modification and the impact of their variations will lay the foundation for a deeper understanding of m6A modification’s functional roles.

Looking ahead, a pivotal direction for m6A research involves the rigorous translation of foundational discoveries and predictive models into tangible clinical applications. This necessitates moving beyond computational predictions to robust experimental validation and clinical trials, ensuring the utility of the identified m6A biomarkers and targeted therapeutic strategies in patient stratification, diagnosis, prognosis, and treatment. In the future, m6A research is expected to play a greater role in precision medicine, drug development [75,76], and the analysis of complex diseases [77,78]. Future research will continue to explore the specific mechanisms of m6A modification, develop more precise and efficient therapeutic strategies, and bring hope to cancer patients.

## Figures and Tables

**Table 1:** Comparison of m6A detection techniques.

Technique	Resolution	Throughput	Advantages	Disadvantages
**MeRIP-seq**	Medium (100–200 nt)	High	Low cost, suitable for whole-transcriptome analysis	Low resolution, difficult to precisely locate single nucleotides
**m6A-CLIP**	High (single-nucleotide)	Medium	High resolution, suitable for studying RNA–protein interactions	Complex experimental procedures, long operation cycles
**miCLIP**	High (single-nucleotide)	Medium	High resolution, precise mapping of m6A modification sites	Complex experimental procedures, high sample quality requirements
**SCARLET**	High (single-nucleotide)	Low	High resolution and specificity	Complex experimental procedures, involves radioisotopes
**scDART-seq**	High (single-nucleotide)	High	Single-cell resolution, revealing intercellular heterogeneity	Complex experimental procedures, high quality and condition requirements
**SMRT Sequencing**	High (single-nucleotide)	Medium	Direct detection of native RNA; long reads allow for isoform-specific m6A profiling and full-length transcript analysis; high accuracy	Lower throughput compared to short-read methods for comprehensive transcriptome-wide profiling; relatively higher cost per base
**Oxford Nanopore Sequencing**	High (single-nucleotide)	High	Direct detection of native RNA; ultra-long reads; real-time data acquisition; simultaneous detection of multiple RNA modifications	Higher raw error rates; challenges regarding development of bioinformatics pipelines for accurate m6A calling and quantification; complex signal processing needed

**Table 2:** Summary of computational tools/software for m6A research.

Tool/Software Name	Main Functionality	Programming Language/Platform	Key Features
m6A-TCPred	m6A site prediction	R language/website platform based on Hyper Text Markup Language (HTML), Cascading Style Sheets (CSS) and Hypertext Preprocessor (PHP), as well as the MySQL tables for metadata storage.	Based on SVM; supports cross-validation and independent testing.
iRNA-m6A	m6A site prediction	Python/web server based on HTML	Utilizes SVM and integrates sequence motifs with bioinformatics attributes, employing pseudo dinucleotide composition.
DeepM6ASeq	m6A site prediction	Python	CNN-BiLSTM architecture for automatic extraction of local features; high predictive accuracy suitable for use with large-scale data.
HSM6AP	High-precision m6A site prediction	Python/web server based on HTML	XGBoost-based ensemble framework integrating sample weighting strategies and multi-dimensional feature fusion; designed for human RNA.
M6APred-EL	m6A site prediction	Python 2.7/web server based on HTML	SVM-based prediction model.
EDLm6APred	m6A site prediction	Python/web server based on HTML	Employs bidirectional LSTMs combined with word embedding algorithms to capture contextual information and long-term dependencies.
M6A-BERT-Stacking	Tissue-specific m6A prediction	Python 3.8.3	Integrates BERT and stacking strategies (ResNet + BiLSTM + BERT) to capture contextual information and enhance overall prediction performance.
SRAMP	m6A site prediction	R language 2.15, Perl 5.8/web server based on HTML	Leverages Random Forest algorithm, based on sequence-derived features; demonstrated high predictive accuracy.
Gene2vec	m6A site prediction	Python 3.6	Combines subsequence embeddings with deep learning using Word2vec-inspired NLP technique to discern contextual patterns.
iRicem6A-CNN	DNA N6-methyladenine prediction	Python/web server	CNN model using dinucleotide one-hot encoding; accounts for nucleotide context to enhance performance in rice DNA.
DeepM6ASeq-EL	m6A site prediction	Python 3	Ensemble learning model combining five LSTM-CNN subnetworks using a hard voting strategy; utilizes diverse sequence context features for human mRNA.
CLSM6A	High-resolution m6A prediction	Python 3/web server based on HTML	Employs multi-layer neural networks to automatically extract single-nucleotide-resolution features.
DLm6Am	m6Am modification recognition	Python 3.7.12/web server	Uses multi-layer neural networks to automatically extract sequence and chemical features; high generalization ability.
DeepM6APred	m6A site prediction	Python 3/web server	Integrates deep feature representations (from deep belief networks) with traditional handcrafted features for enhanced accuracy.

**Table 3:** Performance comparison of common m6A prediction tools.

Model	Testing Method	Availability of Data	Method	Performance				
				Acc	Sn	Sp	MCC	AUC
m6A-TCPred	Cross-validation and independent testing	http://www.rnamd.org/m6ATCPred/ (accessed on 8 July 2025)	SVM	0.801	0.806	0.796	0.603	0.879
iRNA-m6A	10-fold cross-validation test	http://lin-group.cn/server/iRNA-m6A/index.html (accessed on 8 July 2025)	SVM	0.912	0.868	0.956	0.83	0.93
DeepM6ASeq	Five-fold cross-validation	https://github.com/rreybeyb/DeepM6ASeq (accessed on 8 July 2025)	CNN	0.763	0.75	0.73	0.499	0.850
HSM6AP	Five-fold cross-validation test	http://lab.malab.cn/~lijing/HSM6AP.html (accessed on 8 July 2025)	XGBoost	0.953	0.916	0.943	0.651	0.981
M6APred-EL	10-fold cross-validation	https://github.com/chr2117216003/M6APred-EL (accessed on 8 July 2025)	SVM	0.808	0.807	0.810	0.620	0.90
EDLm6APred	Five-fold cross-validation	http://labiip.net/index.php (accessed on 8 July 2025)	RNN	0.786	0.713	-	0.579	0.861
M6A-BERT-Stacking	Five-fold CV and independent test	https://github.com/liqianyue/zeitgeist/tree/master/m6A_BERT_Stacking (accessed on 8 July 2025)	Resnet + BiLSTM + BERT	0.790	0.816	0.764	0.582	0.871
